# Case report: Anti *N*-methyl-D-aspartate autoimmune encephalitis following a mildly symptomatic COVID-19 infection in an adolescent male

**DOI:** 10.3389/fpsyt.2023.1270572

**Published:** 2023-12-04

**Authors:** Thomas Hainmueller, Lambert Lewis, Tzvi Furer

**Affiliations:** ^1^Department of Psychiatry, New York University Langone Medical Center, New York, NY, United States; ^2^Department of Child and Adolescent Psychiatry, Child Study Center at Hassenfeld Children's Hospital of New York at NYU Langone, New York, NY, United States

**Keywords:** case report, psychosis, encephalitis, *N*-methyl-D-aspartate, COVID-19, SARS-CoV2

## Abstract

**Background:**

Antibodies against *N*-methyl-D-aspartate receptors are the most commonly identified cause of autoimmune encephalitis. While predominantly associated with malignancies, cases of anti-*N*-methyl-D-aspartate receptor autoimmune encephalitis have been reported after infections with the herpes-simplex virus or, more recently, in patients with severe COVID-19 disease.

**Case presentation:**

A previously healthy 17-year-old male adolescent acutely developed psychosis with auditory and visual hallucinations, fluctuating mental status, and an isolated seizure 5 weeks after a mildly symptomatic COVID-19 infection. The symptoms continued to worsen, accompanied by catatonia, and additional neurological symptoms developed during the initial antipsychotic treatment. A diagnostic workup revealed antibodies against *N*-methyl-D-aspartate receptors in the cerebrospinal fluid without other major abnormalities. After establishing the diagnosis, initiation of immunomodulatory therapy stopped the symptom progression and led to full recovery within 2 months.

**Conclusion:**

The case is remarkable in that anti-*N*-methyl-D-aspartate receptor autoimmune encephalitis developed shortly after a COVID-19 infection in an adolescent, despite the individual experiencing only mild COVID symptoms. The diagnosis should be considered in cases of acute-onset psychotic symptoms during or after COVID-19 infection, particularly in individuals without a prior psychiatric history, who present with atypical psychiatric or neurological features.

## 1 Introduction

Psychosis can be predominantly associated with psychiatric diseases, but a range of rare and insidious infectious (1, 2), metabolic (3), or autoimmune (4) disorders can produce presentations of psychotic symptoms that are challenging to distinguish from primary mental illness. A recently described (5) form of limbic autoimmune encephalitis (AE), caused by autoantibodies against *N*-methyl-D-aspartate receptors (NMDAR), often presents initially with psychotic symptoms, which then progress to involve neurological manifestations, such as abnormal movements, catatonia, seizures, and autonomic and respiratory dysregulation that can require intensive level care and be lethal if untreated (6). A recent large-scale prospective study among patients admitted for suspected anti-NMDAR AE (7) indicates a wide spectrum of neuropsychiatric manifestations, including psychotic and affective symptoms, catatonia, global cognitive dysfunction, seizures, and dyskinesias. This study further suggests that a fluctuating course of delirium and catatonic features may distinguish cases of anti-NMDA AE from others with similar presentations (7). The treatment of anti-NMDAR AE requires rapid initiation of immunomodulatory therapies, such as intravenous immunoglobulins (IVIG), steroids, rituximab, and cyclophosphamide (4, 8). Anti-NMDAR AE frequently develops as a paraneoplastic syndrome to teratomas of the ovaries, or, more rarely, of the testicles or mediastinum (9–11). It is more prevalent in females, and cases in male patients are rarely associated with tumors (12).

More recent studies indicate that infections with neurotrophic viruses, such as herpes simplex virus, can trigger flares of anti-NMDAR AE (13, 14). The severe acute respiratory syndrome coronavirus 2 (SARS-CoV-2), which causes the COVID-19 respiratory disease, has neurotrophic properties (15). A recent series of case reports in adult (16–18) and pediatric patients (19, 20) indicate that infection with SARS-CoV-2 could induce autoimmunity against NMDAR. While most of these cases were observed in patients hospitalized for severe COVID pneumonia with intensive-level care, the patient described here developed NMDAR AE after a mildly symptomatic COVID-19 disease that could have easily been overlooked in the context of a recent traumatic immigration experience. The case warrants caution both about the complex neuropsychiatric sequela of COVID-19 and vigilance about organic causes for psychiatric symptoms in complex psychosocial settings.

## 2 Case description

The following patient information was de-identified: A 17-year-old male adolescent, who had migrated from Mesoamerica to the United States 6 weeks prior to his initial hospital presentation, was brought to the emergency room after an episode of disorganized and aggressive behavior with delusional thoughts and auditory hallucinations. Multiple collateral sources, including the patient’s mother, with whom he had lived his entire life, confirmed that he had no prior psychiatric or medical history, other than myopia. He had never required or received psychiatric treatment, and there was no pertinent family history of mental illness. Upon entering the United States as an unaccompanied minor in December 2021, he was detained for 4 weeks after crossing the Mexican border. No behavioral abnormalities were noted during this period, but the patient had a fever of 38°C (100.4°F), along with nasal congestion, cough, and headache with positive rapid antigen testing for SARS-CoV-2 and a positive rapid streptococcal antigen test. A chest x-ray presented no abnormalities. He was treated with a five-day course of oral amoxicillin and further received vaccinations against hepatitis A, hepatitis B, human papilloma virus, influenza, meningococcus, measles, mumps, rubella, tetanus, diphtheria, pertussis, polio, and varicella. The patient later reported being held in social isolation for 15 days and experiencing a “change to his mind” along with problems with his memory during this period.

After being released from border detention, the patient went to stay with relatives in the United States. Initially, no behavioral or neurological abnormalities were noted; however, 1 week before his presentation, the patient likely experienced a generalized seizure with symptoms such as tongue biting, loss of consciousness, postictal paresis, and confusion, as later reported by his family. He also started to display intermittent aggression and endorsed auditory hallucinations of voices, which prompted his initial presentation to an outside hospital. The medical workup, including the basic bloodwork, urine toxicology (see Table 1), and a non-contrast computed tomogram (CT) of the head, was unremarkable. He was initially considered to be suffering from a primary psychotic disorder, started on risperidone, and transferred to our care 4 days later.

**Table 1:** Laboratory studies during hospitalization (for tests performed repeatedly, the values closest to the initial presentation are shown).

Type of study	Individual results
Basic blood and serum studies	WBC *7.9 tsd/mcL (5.24–9.74)*, RBC 4.59 *mill/mcL (3.74–4.93)*, HGB *13.7 g/dL (11.0–14.3)*, HCT *40.5% (31.4–41.0)*, PLT *361 tsd/mcL (180–299)*, PT *14.7 s (10.5–13.4)*, INR *1.2 (0.88–1.13)*, aPTT *33.1 s (29.3–35.7)* Sodium *144 mmol/L (136–145)*, Potassium *3.4 mmol/L (3.5–5.0)*, Chloride *103 mmol/L (98–108)*, CO2 *24 mmol/L (22–29)*, BUN *11 mg/dL (8 – 22)*, Creatinine *0.79 mg/dL (0.5–1.3)*, Glucose *124 mg/dL (65–115)*, Calcium *9.8 mg/dL (8.6–10.2)*, Phosphorus *4.8 mg/dL (2.7–4.5)*, Magnesium *1.8 mEQ/L (1.3–1.9)* Albumin *4.9 g/dL (3.5–5.0)*, Total Protein *7.8 g/dL (6 – 8)*, Total Bilirubin *0.6 mg/dL (0.2–2.1)*, Direct Bilirubin *<0.2 mg/dL (0–0.2)*, Alk Phos *152 U/L (40–150)*, ALT (SGPT) *24 U/L (10–41)*, AST (SGOT) *40 U/L (5–40)*, Globulin *2.9 gm/dL (2.5–3.0)*, Hemoglobin A1c *5.6% (4.0–5.6),* Cholesterol *128 mg/dL (<=170)*, HDL cholesterol *43 mg/dL (40–60)*, Triglyceride *60 mg/dL (<=150)* TSH *2.4 uIU/mL (0.35–5.5)*, free T4 *1.54 ng/dL (0.93–1.7)*; ESR *7 mm/h (0–10)*; CRP *1.16 mg/L (0–3)*; B12 *766 pg/mL (200–700)*; Folate *12.12 ng/mL (0–24)*; Acetaminophen *<15 mcg/mL (0–30)*; Salicylates *<3 (15–30)*; Ceruloplasmin *27 mg/dL (15–30)*; ACE *37 U/L (14–82)*; Ethanol *<10 mg/dL (0–10)*; Thyroglobulin *8.95 ng/nL (1.5–59)*; Total carnitine *85 umol/L (27–73)*; free carnitine *58 umol/L (20–55)*; pyruvate *2.2 mg/dL (0.3–1.5).* Plasma acylcarnitine analysis: *Unspecific pattern.* Plasma amino acid analysis: *Unspecific pattern.*
Urine studies	Urine toxicology *negative* for Barbiturates, Benzodiazepines, Cocaine, Methadone, Opiates, THC Urine analysis *negative* for Ketones, Blood, Nitrites, Leucocyte Esterase, and Bacteria; Protein *100 mg/dL*, WBC *2 (0–2)*, RBC *3 (0–4)*; Arsenic* *5.64 mcg/d (<35)*, Mercury* *<2.8 mcg/d (<2)*, Cadmium* *<0.7 mcg/d (<0.7)*, Lead* *1.41 mcg/d (<2)*; Chlamydia PCR *not detected*, Neisseria gonorrhea PCR not detected *24 h excretion approximated using Metal/Creatinine ratio and CKD-EKD estimation of 24 h creatinine. Ref. values for age range > 18 years.
Basic CSF studies	Protein *27.4 mg/dL (15–45)*, Glucose *57 mg/dL (40–80)*, WBC *2–3 cells/mcl (0–5)* Acid Fast Cultures *no growth*, Cryptococcal antigen *negative*, Bacterial culture *no growth*, Fungus / India ink *no growth in 4 weeks*
CSF PCR studies	*Negative* for *Mycobacterium tuberculosis*; *E. coli*, *H. influenzae*, Listeria, *Neisseria meningitidis*, Streptococcus agalacticae, *Streptococcus pneumoniae*, Cytomegaly virus, Herpes simplex virus (HSV) 1, HSV2, Human herpesvirus 6, Human parechovirus, Varicella zoster virus, *Cryptococcus neoformans*/gattii
Serum antibody studies	Serum *non reactive* for: ANA *(<1:80),* Quantiferon, Lyme IgG/IgM *0.12 (0.01–0.89), Treponema Pallidum*, HIV 1,2 AG/Ab by CMIA, Histoplasmosis *<1:8,* Cryptococcal Ag, Trypanosoma cruzi Ab *0.3 IV (<1.0)*, Strongyloides Ab, Cysticercosis Ab *<0.9 (<0.9),* Anti streptolysin O *72 IU/mL (0–199),* Anti-DNAseB titer *134 U/mL (0–170),* Thyroglobulin Ab *<20.0 IU/mL (<40),* Thyroid Peroxidase Ab *<10.0 IU/mL (<34.9)* Mayo PAVAL Serum: *non reactive* for AChR Ganglionic Neuronal Ab, AGNA 1–3, CRMP-5-IgG, Neuronal (V-G) K+ Channel Ab, P/Q-Type Calcium Channel Ab, PCA 1,2,Tr Hepatitis A IgG *reactive* IgM *non-reactive*, Hepatits B surface Ab *reactive*, core AB *non-reactive,* Hepatitis C Ab *non-reactive 0.11 S/CO (0–0.99)*
CSF antibody studies	Mayo ENC1 CBA CSF: reactive for NMDAR Ab*, *non-reactive* for LGI1-IgG, CASPR2-IgG, GAD65 Ab, GABA-B-R Ab, AMPA-R Ab, Anti-Neuronal Nuclear Ab, Type 1–3, Anti-Glial Nuclear Ab, Type 1; Purkinje Cell Cytoplasmic Ab, Type 1, 2, Tr; Amphiphysin Ab; CRMP-5-IgG; DPPX Ab IFA; GFAP IFA; mGluR1 Ab IFA
Other tests	SARS-CoV-2 PCR nasal swab (positive on initial assessment, negative since second day of hospitalization),

*Antibody detected when tested undiluted by cell-based assay. Reflex at 1:2 on tissue-based assay non-reactive. NMDAR Ab positive, “N-methyl-D-Aspartate Receptor Antibody.

### 2.1 Clinical findings

**Figure 1:**
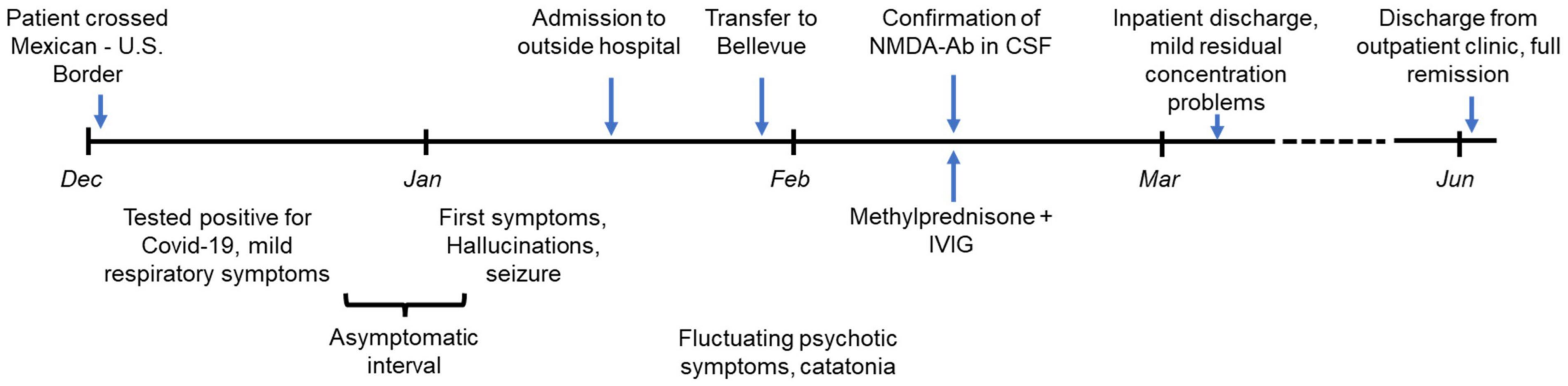
Timeline of relevant events. IVIG, intravenous immunoglobulins.

During our initial evaluation, the patient displayed intense and labile affect and periods of confusion, loss of orientation to time, location, and situation, with aggressive behavior. He voiced subjective complaints about problems with his ‘nerves’ and ‘memory.’ He appeared internally preoccupied and reported both visual and auditory hallucinations. Additionally, he exhibited significant paranoia, believing that individuals were pursuing him with an attempt to physically and sexually assault him. These manifestations lasted for hours at a time and were interleaved with periods of linear thought process and appropriate thought content. The presentation appeared most consistent with delirium with psychotic features, although the etiology was unknown at the time. A primary psychotic disorder was considered as a differential diagnosis and had initially been suspected by the referring hospital. However, this seemed less likely on our assessment given the absence of any psychiatric history and the strong fluctuance of symptoms. The initial neurological examination performed while the patient was fully oriented and with organized thought process revealed deficits in attention and delayed recall, but no focal neurological deficits were identified. In the later hospital course, 2–3 weeks after admission (Figure 1), the patient further developed hypersomnolence and fluctuating catatonic features, including verbigeration, stupor, minimal verbal responsiveness, staring, posturing, negativisms, waxy flexibility, *Mitgehen*, and mild autonomic instability [Bush-Francis score (21) up to 23]. In addition, he started to show neurological symptoms, specifically dysarthria and gait ataxia.

**Figure 2:**
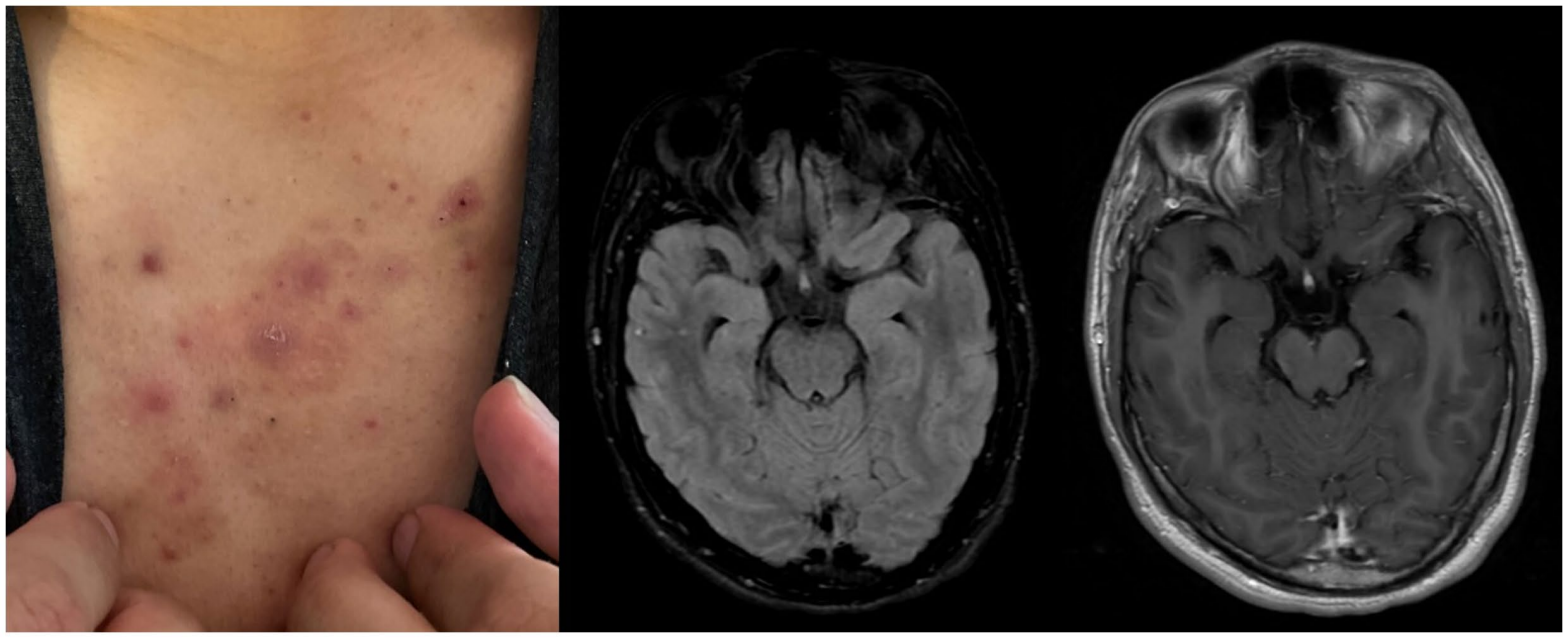
**(A)** Maculopapular rash (chest). **(B)** Ax FLAIR (T2) pre contrast, no abnormal signal enhancement. **(C)** Post contrast AxT1 FLAIR, no abnormal contrast enhancement.

During the physical exam, a maculopapular rash was observed on the patient’s chest and back (as shown in Figure 2A). This rash had started to develop approximately 2–3 weeks prior to the initial presentation. Magnetic resonance imaging with gadolinium contrast (Figures 2B,C) did not show any structural abnormalities or abnormal intracranial enhancement. An electroencephalogram with video observation was discontinued by the patient after 6 h and showed very mild diffuse polymorphic slowing with no evidence for focal cerebral dysfunction or epileptiform activity. Cerebrospinal fluid (CSF) revealed normal cell counts, protein, and glucose levels (Table 1), but later tested positive for autoantibodies against NMDA receptors. Further infectious, metabolic, and autoimmune serum and CSF studies provided no significant findings (Table 1). CT studies of the chest and abdomen, along with a testicular ultrasound, did not indicate any underlying malignancy.

### 2.2 Treatment and outcomes

Initial treatment attempts with Risperidone, started in the outside hospital and later switched to Chlorpromazine upon admission to our unit, resulted in modest improvements of agitation without major changes in psychotic symptoms (Table 2). Due to the reported occurrence of a generalized seizure prior to hospitalization, valproic acid was added to the treatment regimen in the first week of admission and continued throughout the hospitalization. The patient remained seizure-free for the remainder of the follow-up period. Lorazepam was added after the development of catatonic symptoms 3 weeks into the hospitalization, resulting in improvements in catatonia (Table 2). However, residual verbigeration, posturing, rigidity, negativisms, and verbal paucity persisted. These symptoms continued to fluctuate and were not always present at the same time. The patient did not tolerate increases in the dose of Lorazepam above 1.5 mg three times daily due to excessive sedation. CSF studies were considered early in the hospital course, but the results were delayed for a number of logistical reasons. The presence of autoantibodies against NMDAR in the CSF was confirmed 3 weeks into the hospital course, and the treatment with methylprednisolone, IVIG, and rituximab was initiated and tolerated without major side effects. This led to significant improvements in neurological symptoms, behavioral control, and organization of his thought process, which enabled us to discharge the patient 5 weeks later with near-total recovery from his baseline mental functioning.

**Table 2:** Treatment attempts and responses.

Drug	Dosage	Time and duration	Response
Risperidone	0.5 mg twice daily	Days 1–2 of admission	Symptoms unchanged
Chlorpromazine	50 mg twice daily	Week 1–2	Modest improvements in agitation.
Sodium valproate	500 mg every 12 h	Week 1–8	No overt seizures throughout admission.
Lorazepam	1–1.5 mg 2–3 times daily	Week 3–8	Improvements in Bush-Francis score (23 to 12) but residual catatonic symptoms (see text).
Methylprednisolone	1 g i.v. daily	Week 4 for 5 days	No further progression of neurological symptoms and reduction in agitation. Restoration of thought process
Intravenous (i.v.) immunoglobulins	100 g i.v. daily	Week 4 for 2 days	
Rituximab	1 g i.v. weekly	Week 4–5	
Olanzapine	5 mg nightly	Week 5–8	Reduction in agitation

The patient was monitored for 3 more months in an outpatient setting. Olanzapine, lorazepam, and valproic acid were briefly continued after the patient was discharged from the hospital with a plan to gradually taper but then self-discontinued by the patient and his family. The patient’s psychiatric and neurological status continued to remain at his personal baseline for several weeks despite discontinuation of all medications and therefore no attempts were made to re-initiate them. He could be discharged from the outpatient clinic without any residual psychiatric or neurological symptoms in June 2022 (Figure 1).

## 3 Discussion

The present case raises a few points of clinical and academic importance: First, early symptoms of AE are often predominantly psychiatric, while neurological symptoms such as orofacial automatisms, motor anomalies, and seizures may develop only later in the course of the disease (22). This can cause detrimental delays in diagnosis and adequate treatment. In this particular case, psychosocial factors, such as trauma during detainment and separation from the family, can easily distract from the underlying diagnosis. However, the rapid onset of a psychotic syndrome with atypical features, such as visual hallucinations and intermittently altered mental status in a patient without prior psychiatric history, provides valuable guidance toward an underlying organic cause (4). Recent studies indicate that the symptoms displayed by the patient, namely delirium with psychotic features and catatonia, are indeed highly suggestive and characteristic of anti-NMDAR AE (7).

In cases like the present one, there should be no hesitation to assess for autoantibodies in serum and CSF, which was unfortunately delayed in this case. International consensus guidelines for pediatric anti-NMDAR AE suggest that the initiation of immunomodulatory treatment is crucial for clinical improvement and may be initiated even before laboratory confirmation of anti-NMDAR antibodies, especially if clinical symptoms are convincing and alternative diagnoses have been reasonably excluded (8), as was the case here. Recent studies further indicate that allosteric modulation of NMDAR may be a promising complementary treatment approach, particularly in refractory cases (23).

Second, an emerging association between the COVID-19 disease and NMDAR AE, if further substantiated in the future, should prompt differential diagnostic consideration in cases of acutely emerging psychiatric symptoms following after a recent SARS-CoV-2 infection as present in this case. While the association in this case does not imply causality, the temporal association between COVID-19 symptoms and development of anti-NMDAR AE in this and other cases (16–20), along with the known neurotropic properties of the SARS-CoV-2 virus (15, 24), suggests that autoimmunity could develop in response to exposure of neuronal antigens in the setting of acute infection, with a delayed onset of AE symptoms over the following weeks. In contrast to other reported cases of NMDAR AE after COVID-19 infection (17, 18), the patient reported here developed these symptoms in the absence of significant COVID symptoms and after a completely asymptomatic period of 2–3 weeks following full convalescence from COVID.

The key takeaway from the current case is that AE should be considered in cases of sudden-onset psychiatric symptoms in patients, even after mild or asymptomatic COVID-19 infection. This consideration is particularly crucial if there is no prior psychiatric history or if patients present with atypical symptoms such as fluctuating mental status or comorbid neurological symptoms.

## 4 Patient perspective

“I’m feeling fine now. Before, everything was making me anxious. I cannot remember much about the time in the hospital but now things are normal again.”

The patient’s assessment describes both the psychotic and affective elements of his experience, leading up to hospitalization, as well as his rapid recovery after successful treatment. He reported ‘memory problems’ and exhibited impaired delayed recall early in his hospitalization. His report of amnesia for the hospitalization period is in line with the involvement of the limbic system in anti-NMDA AE.

## Data availability statement

The original contributions presented in the study are included in the article/supplementary material, further inquiries can be directed to the corresponding author.

## Ethics statement

Written informed consent was obtained from the individual(s), and minor(s)’ legal guardian/next of kin, for the publication of any potentially identifiable images or data included in this article.

## Author contributions

TH: Conceptualization, Investigation, Writing – original draft, Writing – review & editing. LL: Conceptualization, Supervision, Writing – review & editing. TF: Conceptualization, Supervision, Writing – review & editing.
